# Intention to Use Automated Diagnosis and Clinical Risk Perceptions Among First Contact Clinicians in Resource-Poor Settings: Questionnaire-Based Study Focusing on Acute Burns

**DOI:** 10.2196/56300

**Published:** 2025-06-03

**Authors:** Constance Boissin, Lisa Blom, Zara Taha, Lee Wallis, Nikki Allorto, Lucie Laflamme

**Affiliations:** 1 Department of Medical Epidemiology and Biostatistics, Karolinska Institutet, Stockholm, Sweden; 2 Department of Global Public Health, Karolinska Institutet, Tomtebodavagen 18A, Widerstromska Huset, Stockholm, 17177, Sweden, 46 852480000; 3 Division of Emergency Medicine, Faculty of Health Sciences, University of Cape Town, Cape Town, South Africa; 4 Pietermaritzburg Burn Service, University of Kwa-Zulu Natal, Durban, South Africa; 5 Institute for Social and Health Sciences, University of South Africa, Pretoria, South Africa

**Keywords:** artificial intelligence, burns, clinical decision support systems, clinical risk perception, diagnosis, computer-assisted, first contact clinicians, image-based diagnostics, intention to use, telemedicine, triage

## Abstract

**Background:**

Burn automated diagnosis may be instrumental for accurate and timely decision-making at point-of-care, helping to ensure that the right patients are triaged to burns centers. This is particularly important in resource-poor settings.

**Objective:**

We studied the intention of nonspecialized clinicians to engage in automated diagnosis in burn care as well as their perceptions toward clinical risks.

**Methods:**

A self-administered survey was used among a purposive sample of first contact clinicians (n=56) and burns specialists (n=35). The survey had 2 main parts: 1 measuring the intention to use automated diagnosis as per 7 constructs of the Automation Acceptance Model (yielding 8 hypotheses) and 1 on clinical risk perceptions (likelihood and severity of 7 risks). Structural Equation Modelling was used to test the hypotheses among first contact clinicians, and the Mann-Whitney U test was used to measure differences in risk perceptions between the two clinical groups.

**Results:**

Many first contact clinicians would intend to use automated diagnosis for burns should the technology be made available in their departments (41/56, 73%). The Automation Acceptance Model concepts contributed moderately to explain what the intention to use automated diagnosis rests on (*R*^2^=0.432), with 5 out of 8 hypotheses being supported. The intention to use automated diagnosis was associated with perceived usefulness but not with attitudes toward using it. Of the 7 risks studied, the 1 that was most often considered as high risk of occurring was that of complex burns not being recognized (n=23, 29%). The 2 groups differed significantly in their concern regarding both the likelihood of happening and the severity of 2 risks: the undermanagement of severe burns and the overmanagement of minor burns. Specifically, a larger proportion of first contact clinicians were more concerned than burns specialists (n=13, 27% versus 6% and n=11, 23% versus 6% for undermanagement and overmanagement, respectively).

**Conclusions:**

Almost three-quarters of first contact clinicians were inclined to seek automated advice for burn diagnosis. The proposed model contributes to explaining the intention to use with 5 hypotheses supported. When seeking additional determinants, clinical risk perception is a dimension that should be considered in any artificial intelligence implementation process, to help ensure sustainability.

## Introduction

Image-based automated diagnosis is developing rapidly, not least with the access to deep neural networks and even more with recent methods that can improve performance, such as foundation models or vision transformers [1-3]. These algorithms can outperform clinicians in medical specialties such as cancer pathology [4], ophthalmology [5], dermatology [6], and burns [7]. However, implementation of the technology into the clinical setting has been relatively low and is dependent on the medical specialty. For instance, results in the radiology field emphasized the need for grounded, well-structured, and hospital-wide implementation processes [8]. In surgical pathology, rapid and accurate results provided by automated diagnosis enabled pathologists to focus on more complex consultative tasks [9]. Further, maxillofacial surgeons, ophthalmologists, dermatologists, and radiation oncologists acknowledged the need to be trained before implementation and to have well-defined responsibilities among all users involved in the implementation process, especially if errors were to happen [10,11].

In Sub-Saharan Africa, the potential for artificial intelligence (AI) is big, as it could assist with early detection of infectious diseases [12,13], but also assist with the lack of specialists in underserved areas [14]. As an example, a solution for tuberculosis detection in chest x-rays has been proposed in South Africa [15].

Despite those promises, AI has not unleashed its full potential in the continent, with the uptake of this technology being relatively low at first point of care, perhaps because of limited involvement from health care specialists at the development stage [16,17]. Users’ trust and task seamlessness [18] have been well described as uptake barriers since the introduction of telemedicine [19,20] and mHealth [21]. Gaining knowledge on first contact clinicians’ intention to adopt automated diagnosis procedures is an essential milestone in their development and implementation process, allowing for user-sensitive transformations of diagnostic decision making in the clinical setting.

The visual nature of burns, the wide uptake of mHealth [22], the progress made in the technology [16], and the scarcity of specialists, not least in low resource settings [23], are important arguments in favor of implementing automated diagnosis in this clinical domain. This current study follows prior work showing that the technology performs well [16] and investigates the factors influencing the willingness of first contact clinicians to use automated diagnostic assistance in burn care. It adds to the body of knowledge generated from implementation science studies, setting the stage for translation from knowledge to practice and informing on end users’ perceptions in view of a change in clinical procedures.

## Methods

### Aim

The primary aim of this study was to determine the factors influencing the intention to use an automated burn diagnosis tool among first contact clinicians. An additional aim was to assess whether the views of those clinicians are like those of burns specialists, in particular, how they perceive the likelihood and severity of certain clinical risks.

### Study Design

This was a questionnaire-based cross-sectional study. The CHERRIES checklist for reporting results of e-surveys was used [24], with results presented in Table S1 in Multimedia Appendix 1.

### Data Collection

In 2019, two consecutive international congresses took place in South Africa and gathered clinicians involved in burn injury care at different levels, both first-contact care and specialized care delivery. Those were judged good opportunities to reach out to several professionals from different parts of Sub-Saharan Africa and other parts of the world that, otherwise, would have been difficult to reach. The survey took place in the context of the development of an algorithm for image-based automated burn diagnosis [23]. The meetings were the Pan African and South African Burn Congress 2019 and the EMSSA (7th Emergency Medicine Society of South Africa) International Conference. At both events, participants were informed about this study taking place when they registered, and pamphlets containing information about the survey were distributed in sessions. Using a QR code provided, those who wished to participate could go on and fill in the survey on their own device or using one of the tablets provided by the research team.

### Questionnaire

A questionnaire developed built on previous publications [20,21,25] was used to assess the intention to use an automated burn diagnosis tool among the participants based on the Automation Acceptance Model (AAM) [25]. For that purpose, the questionnaire was divided into 3 parts. Following some information on the context of automated burn diagnosis tool development, and with a specific description of what such a tool would consist of, the first section included demographic questions about the participant and its practice in burn care. The second part included 23 questions representing the 7 constructs from AAM [25], as well as anxiety [21,26,27] to reflect the intention to use an automated burn diagnosis tool in resource-poor settings in Africa (Table 1). The 7 constructs were behavioral intention (1 measurement item), attitude (3 items), perceived usefulness (8 items), perceived ease of use (2 items), trust (2 items), and compatibility (2 items) all directly inspired from the AAM and adapted to burns diagnosis and anxiety (5 items) which was shown to be predictive in similar populations for the introduction of telehealth. All constructs were measured on a 7-point Likert scale from 1 “strongly disagree” to 7 “strongly agree.” The third part of the questionnaire evaluated the participant’s perceptions of the risks linked with the use of an automated burn diagnosis tool. Seven risks were specifically evaluated: (1) that severe burns are missed, leading to undermanagement; (2) that minor burns are diagnosed as severe, leading to overmanagement; (3) that clinicians feel less responsible for the care they provide; (4) that the emotional well-being of the patient will be neglected; (5) that the patient confidentiality will be breached; (6) that patients will be less empowered in decisions around their care; (7) that some complex burns may not be recognized by the algorithm. For each risk, the participant had to evaluate the likelihood of happening on a 3-point scale from low or very low to high or very high; and the severity of the risk would it happen on a 4-point scale (insignificant, minor, moderate, or major).

**Table 1.** Measurement items used in the questionnaire in relation to the defined construct they measure.

Construct (definition)	Measurement items
Behavioral intention: The motivational factors that determine an individual’s readiness to use the technology, which is assumed to be an immediate antecedent of behavior [28]	· I intend to use automated diagnosis for burns as soon as it becomes available in my department
Attitude: An individual’s positive or negative feelings about using the technology [28]	· I believe that using automated diagnosis for burns is a good idea· Using automated diagnosis for burns would make work more interesting· I believe that using automated diagnosis for burns is a bad idea
Perceived usefulness: The degree to which an individual believes using the technology will assist in performing a task [29]	· It would make it easier to do my job when managing patients with burns· It could improve the care I give to my patients with burns· It will reduce the probability of medical errors in burn care· Using it will improve the quality of my burn-related work· It will be useful in my burn-related work· It will be useful in improving patient safety· I would use it for burn wounds that I normally would hesitate to consult a specialist about· Using it in my burn-related work would make me feel more independent
Perceived ease of use: The degree to which an individual believes using the technology will be effortless [29]	· Learning to operate a tool like this would be easy for me· It would be easy for me to become skilled at using such a tool
Trust: The generalized expectancy to which an individual can rely on the technology [25,30]	· I believe that the diagnostic ability of automated diagnosis is superior to the clinical experience of human doctors· I would have difficulties trusting an automated diagnosis
Compatibility: The degree to which the technology is believed to be consistent with an individual’s existing values, needs, and experiences [25]	· Using a tool like this would fit well with the way I like to work· Using an app like this would be compatible with most aspects of my work
Anxiety: The degree to which emotional reactions are evoked when using the technology [27]	· I feel apprehensive about using such a tool· Using automated diagnosis for burns would make me feel uncomfortable· I am concerned about possible liability issues associated with its use· Working with such a tool would make me feel anxious· Using it would make me feel less anxious about dealing with burns patients

The questionnaire was pilot-tested before the data collection to ensure comprehensibility, content, and length of time.

### Study Population

#### Overview

Two study groups were included in this study, all of whom were familiar with cases from Sub-Saharan Africa: first contact clinicians, as our focus, and burn specialists, as a basis of comparison.

#### First Contact Clinicians

First contact clinicians treating burn injuries at the front line in Sub-Saharan African countries were targeted because they would be the ones using such an image-based automated diagnostic assistance in burn care. The inclusion criteria were therefore that the participant was a doctor or a nurse who may need regular or occasional advice for referral, diagnosis, or treatment of burn injuries in their practice. Another eligibility criterion was that one had to work in an emergency or trauma center (where patients with acute burns are brought) or at a specialized surgical unit that manages patients with burns. Furthermore, the participant had to be practicing, or have practiced in a Sub-Saharan African country.

#### Burn Specialists

Those who took part in this study were burn specialists who have an expert role in their setting when it comes to burns assessment and treatment; they completed a limited part of the questionnaire (see below), where they were asked about potential risks that may accompany the use of automated diagnosis for burns.

A total of 122 participants started the survey, of which 31 were excluded because: they did not complete the consent form (n=6), they did not complete the survey (n=18), they only practiced in a high income country (n=3), or they were prehospital paramedics (n=4). The analyses were performed on the remaining 91 participants.

### Data Analyses

#### Intention to Use Automated Diagnosis for Burn Care

To explain the intention to use automated diagnosis for burn care, 8 hypotheses (presented in Table 2) were proposed based on the 7 constructs previously defined. To evaluate the accuracy and consistency of the developed measurement items with regard to their respective constructs, Cronbach α and composite reliability were measured. Any constructs that had a Cronbach α lower than 0.7 or a composite reliability value lower than 0.6 were excluded from further analyses [31]. The results are presented in Table 3 and show that all constructs are consistent and reliable except for trust, for which the 2 measurement items do not yield an appropriate Cronbach α. This construct was therefore excluded from further analyses and the model.

**Table 2.** Hypotheses tested, definitions, and results obtained.

Hypothesis	Definition	Supported by the results
1	Perceived compatibility is positively related to the perception of the usefulness of automated diagnosis for burn care.	Yes
2	Perceived compatibility is positively related to the perception of the ease of use of automated diagnosis for burn care.	Yes
3	Perceived ease of use of automated diagnosis for burn care is positively related to the perception of its usefulness.	No
4	Perceived usefulness of automated diagnosis for burn care is positively related to attitudes toward using it.	Yes
5	Perceived ease of use of automated diagnosis for burn care is positively related to attitudes toward using it.	No
6	Anxiety is negatively related to attitudes toward using automated diagnosis for burn care.	Yes
7	Perceived usefulness of automated diagnosis for burn care is positively related to the behavioral intention to use it.	Yes
8	Positive attitudes toward using automated diagnosis for burn care are positively related to the behavioral intention of using it.	No

**Table 3.** Constructs summary (number of items and mean), consistency statistic (Cronbach α), and composite reliability measure.

Construct	Number of items	Mean (SD)	Cronbach α	Composite reliability
Behavioral Intention	1	5.4 (1.6)	—^a^	—
Attitudes	3	5.4 (1.1)	0.732	0.846
Perceived usefulness	8	5.3 (1)	0.918	0.934
Perceived ease of use	2	5.7 (1.2)	0.866	0.937
Trust	2	4 (1)	−0.658	0.332
Compatibility	2	5.4 (1.3)	0.886	0.946
Anxiety	5	4.7 (1.1)	0.701	0.803

a Not applicable.

**Figure 1.**
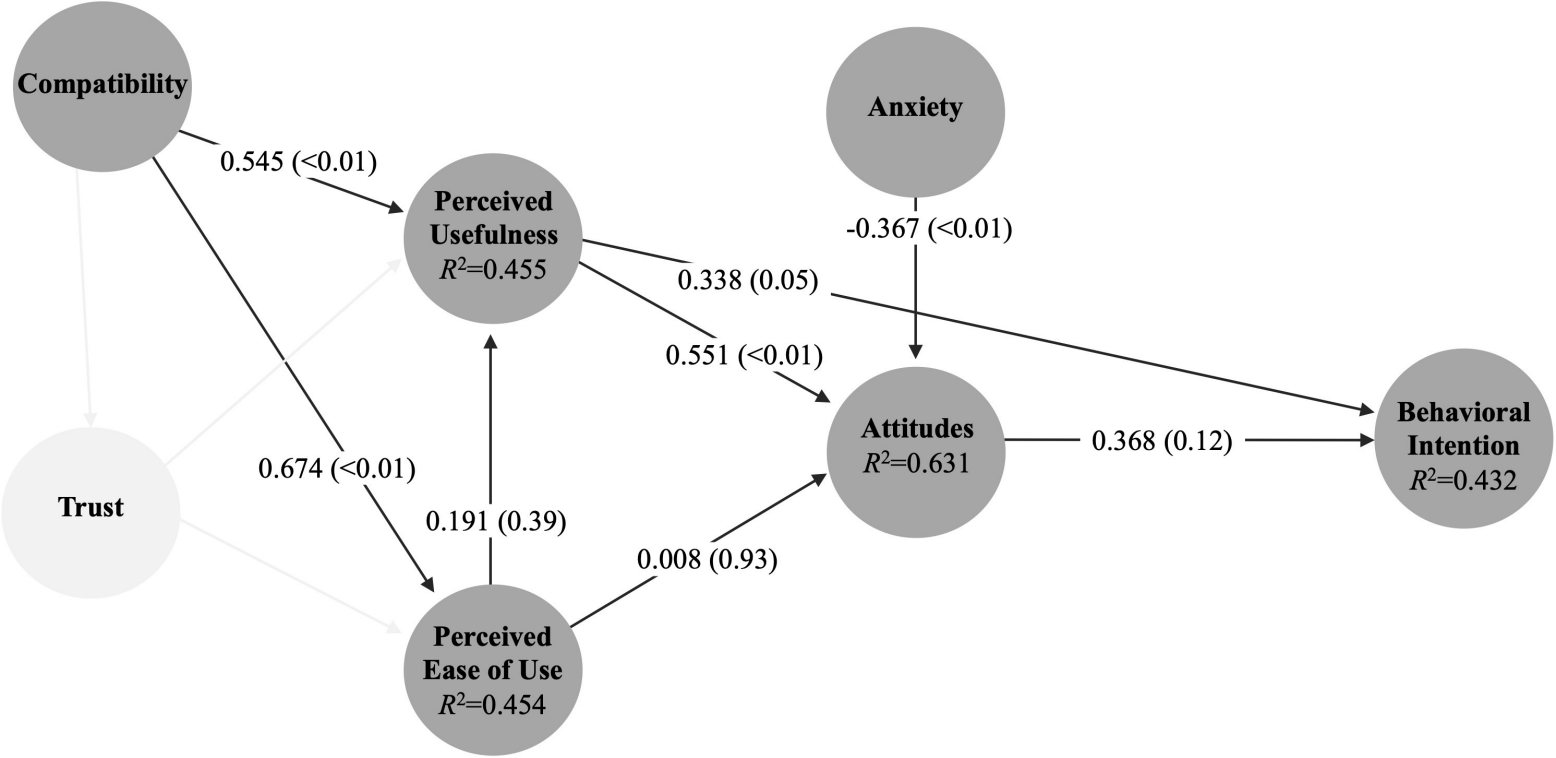
Seven hypotheses generated by the obtained AAM model and their respective path coefficients. AAM: Automation Acceptance Model.

We then used Partial Least Squares Structural Equation Modelling to examine the proposed model (Figure 1) and test the hypotheses. Partial Least Squares Structural Equation Modelling is a multivariate statistical analysis that allows for testing models with an established theoretical foundation and with latent variables [32]. Bias-corrected and accelerated bootstrapping using 5000 subsamples was used to measure statistical significance. We used a hard cutoff to define whether a hypothesis was supported or not, which was an obtained value higher than 0.2 with a *P* value <.05 [20]. Analyses were performed using SmartPLS (version 4.0).

#### Risks That May Occur With Implementation

Both first contact clinicians and burns specialists completed the survey section on potential risks. For each of these risks, the proportion of answers was reported in 3 categories. For the severity of each risk, the categories of insignificant or minor risk were combined and collapsed into one. A nonparametric Mann-Whitney U test was used to assess the differences in opinions between the 2 groups. For that, all data points for the 2 groups are ranked in ascending order, and the sum of the ranks ($R_{1}$ and $R_{2})$ are then calculated for both groups separately (first contact clinicians and burns specialists). The U statistic is then calculated for each group:

$U_{1}=n_{1}∙n_{2}+\frac{n_{1}\left(n_{1}+1\right)}{2}-R_{1}$ for group 1 and $U_{2}=n_{1}∙n_{2}+\frac{n_{2}\left(2+1\right)}{2}-R_{2}$ for group 2

where $n_{1}$ and $n_{2}$ are the sizes of the 2 groups. The smaller U value is then used to assess significance with a defined *P* value threshold of .05.

All analyses were performed using Stata BE (version 17.0; StataCorp LLC).

### Ethical Considerations

This study was approved in South Africa by the Human Research Ethics Committee of the University of Cape Town (Dnr 823/2019) and in Sweden by the Ethics Committee (Etikprövningsmyndigheten [Dnr 2019‐05122]). Informed consent was provided by participants prior to filling in the online survey. The data are deidentified and no compensation was provided for participating.

## Results

Of the 91 respondents who completed at least 1 section of the survey, 62% (56/91) were first contact clinicians and 38% (35/91) were burn specialists. Table 4 presents the respective characteristics of the 2 groups with regard to their demographics and experience with burn care. Overall, these were mostly men (49/91, 54%), although the opposite was observed at first contact care, with over half being women (31/56, 55%). A total of 52% (29/56) of first contact clinicians managed patients with burns at no or few occasions per week, whereas 20% (7/35) of burns specialists manage patients at least once a week.

**Table 4.** Characteristics of participants by clinical expertise.

Characteristic	All (n=91), n (%)	First contact clinicians (n=56), n (%)	Burns specialists (n=35), n (%)
Sex			
Men	49 (53.9)	25 (44.6)	24 (68.6)
Women	42 (46.2)	31 (55.4)	11 (46.2)
Age (in years)			
<35	22 (24.2)	14 (25)	8 (22.9)
35‐39	27 (29.7)	21 (37.5)	6 (17.1)
40‐44	21 (23.1)	15 (26.8)	6 (17.1)
≥45	21 (23.1)	6 (10.7)	15 (42.9)
Occupation			
Nurse	17 (18.7)	13 (23.2)	4 (11.4)
Medical officer	20 (22)	12 (21.4)	8 (22.9)
Registrar	21 (23.1)	16 (28.6)	5 (14.3)
Specialist	33 (36.7)	15 (26.8)	18 (51.4)
Experience with burn treatment			
None or minimal	18 (19.8)	17 (30.4)	1 (2.9)
Moderate	55 (60.4)	34 (60.7)	21 (60)
Extensive	18 (19.8)	5 (8.9)	13 (37.1)
Frequency of managing patients with burns			
No or few occasions	27 (31.9)	29 (51.8)	0 (0)
A few per month	52 (55)	22 (39.3)	28 (80)
A few per week or daily	12 (13.2)	5 (8.9)	7 (20)
Confident in own burn management			
Not at all	4 (4.4)	4 (7.1)	0 (0)
Somewhat	55 (60.4)	39 (69.6)	16 (45.7)
Very	32 (35.2)	13 (23.2)	19 (54.3)

A total of 41 of the 56 (73%) first contact clinicians replied that they agreed or strongly agreed that they would use automated diagnosis for burns if it becomes available in their department. Figure 1 presents the results of the tested model for path coefficients. While perceived usefulness was positively and significantly related to the intention to use, attitudes toward the practice were not. Together, these 2 constructs explain 43% of the variance in intention to use for clinicians at the point-of-care. Almost two-thirds of the variance in attitudes toward the practice (*R*^2^=0.631) could be explained by the 3 constructs of anxiety, perceived usefulness, and perceived ease of use. For its part, compatibility was positively related to the perception of both usefulness and ease of use. Overall, out of the 8 tested hypotheses, 5 were supported in the model (Table 2).

Table 5 indicates the position of the 2 groups of professionals concerning the likelihood of different risks. All respondents aggregated, the risk of complex burns not being recognized and that of clinicians feeling less responsible for the care they provide are the 2 risks that most often were considered as high or very high (23/79, 29% and 19/85, 22%, respectively). There are 2 risks where the opinions of the 2 groups differ significantly, and they concern the undermanagement of severe burns and the overmanagement of minor burns. In both instances, the risk is seen as high or very high by more first contact clinicians than burn specialists.

**Table 5.** Likelihood of each risk to happen as perceived by clinicians at first contact and burn specialists.

Risks assessed for likelihood	Do not know, n	Low or very low, n (%)	Moderate, n (%)	High or very high, n (%)	Mann-Whitney *P* value
That severe burns are missed, leading to undermanagement					.03
First contact clinicians	5	15 (31.3)	20 (41.7)	13 (27.1)	
Burn specialists	0	16 (45.7)	17 (48.6)	2 (5.7)	
All participants	5	31 (37.4)	37 (44.6)	15 (18.1)	
That minor burns are diagnosed as severe, leading to overmanagement					<.01
First contact clinicians	4	14 (28.6)	24 (49)	11 (22.5)	
Burn specialists	1	21 (61.8)	11 (32.4)	2 (5.9)	
All participants	5	35 (42.2)	35 (42.2)	13 (15.7)	
That clinicians feel less responsible for the care they provide					.11
First contact clinicians	3	25 (50)	11 (22)	14 (28)	
Burn specialists	0	23 (65.7)	7 (20)	5 (14.3)	
All participants	3	48 (56.5)	18 (21.2)	19 (22.4)	
That the emotional well-being of the patient will be neglected					.98
First contact clinicians	1	35 (67.3)	11 (21.2)	6 (11.5)	
Burn specialists	1	23 (67.7)	7 (20.6)	4 (11.8)	
All participants	2	58 (67.4)	18 (20.9)	10 (11.6)	
That the patient confidentiality will be breached					.38
First contact clinicians	3	31 (62)	16 (32)	3 (6)	
Burn specialists	0	20 (57.1)	8 (22.9)	7 (20)	
All participants	3	51 (60)	24 (28.2)	10 (11.8)	
That patients will be less empowered in decisions around their care					.22
First contact clinicians	2	35 (68.6)	10 (19.6)	6 (11.8)	
Burn specialists	1	27 (79.4)	6 (17.7)	1 (2.9)	
All participants	3	62 (72.9)	16 (18.8)	7 (8.2)	
That some complex burns may not be recognized by the algorithm					.2
First contact clinicians	8	6 (13.3)	24 (53.3)	15 (33.3)	
Burn specialists	1	8 (23.5)	18 (52.9)	8 (23.5)	
All participants	9	14 (17.7)	42 (53.2)	23 (29.1)	

Table 6 presents the perceived severity of each risk for both groups of respondents. For all risks, the severity is perceived as major to a higher proportion of first contact clinicians than burn specialists. Just as for the likelihood, all respondents aggregated, the risk for complex burns not being recognized is the risk for which severity is most often considered as major (45/82, 55%). A large proportion of clinicians (46/85, 54%) also rated as major the severity of the risk for severe burns being missed, although this is true especially for those at point of care (32/51, 63%). While almost 80% (27/34) of burns specialists considered the severity of minor burns being diagnosed as minor or insignificant, only 40% (21/52) of first contact clinicians did.

**Table 6.** Severity of each of the risks would it be perceived by first contact clinicians and burn specialists.

Risks assessed for severity	Do not know n	Insignificant or minor,n (%)		Moderate,n (%)		Major,n (%)		Mann-Whitney*P *value	
That severe burns are missed, leading to undermanagement								.02	
First contact clinicians	2	6 (11.8)		13 (25.5)		32 (62.8)			
Burn specialists	1	11 (32.4)		9 (26.5)		14 (41.2)			
All participants	3	17 (20)		22 (25.9)		46 (54.1)			
That minor burns are diagnosed as severe, leading to overmanagement								<.01	
First contact clinicians	1	21 (40.4)		24 (46.2)		7 (13.5)			
Burn specialists	1	27 (79.4)		7 (20.6)		0 (0)			
All participants	2	48 (55.8)		31 (36.1)		7 (8.1)			
That clinicians feel less responsible for the care they provide								.07	
First contact clinicians	2	17 (33.3)		17 (33.3)		17 (33.3)			
Burn specialists	1	17 (50)		11 (32.4)		6 (17.7)			
All participants	3	34 (40)		28 (32.9)		23 (27.1)			
That the emotional well-being of the patient will be neglected								.14	
First contact clinicians	4	19 (38.8)		17 (34.7)		13 (26.5)			
Burn specialists	1	19 (55.9)		9 (26.5)		6 (17.7)			
All participants	5	38 (45.8)		26 (31.3)		19 (22.9)			
That the patient confidentiality will be breached								.05	
First contact clinicians	1	21 (40.4)		12 (23.1)		19 (36.5)			
Burn specialists	1	20 (58.8)		8 (23.5)		6 (17.7)			
All participants	2	41 (47.7)		20 (23.3)		25 (29.1)			
That patients will be less empowered in decisions around their care								.03	
First contact clinicians	3	22 (44)		18 (36)		10 (20)			
Burn specialists	1	23 (64.7)		11 (32.4)		1 (2.9)			
All participants	4	44 (52.4)		29 (34.5)		11 (13.1)			
That some complex burns may not be recognized by the algorithm								.096	
First contact clinicians	3	7 (14)		11 (22)		32 (64)			
Burn specialists	3	4 (12.5)		15 (46.9)		13 (40.6)			
All participants	6	11 (13.4)		26 (31.7)		45 (54.9)			

## Discussion

### Main Findings

This study is an attempt to bridge the gap between what is known about the potential performance of automated diagnosis for burn care and the readiness of first contact clinicians to endorse this as a practice. We showed that a majority of first contact clinicians from resource-poor African settings would be willing to use automated diagnosis for burns should the technology be made available in their departments. The concepts embedded in the AAM contributed moderately to explaining what the intention to use such a technology rests on. Of special interest is the association between the intention to use and perceived usefulness, but not with attitudes toward the technology. It is also of note that 5 of the 8 hypotheses derived from the model were supported.

When reflecting upon the risks that such technologies could imply, first contact clinicians showed more concerns than burns specialists. They were typically concerned with the consequences of poor diagnostic accuracy (ie, undermanagement of severe burns and overmanagement of minor burns) and, like their specialized peers, feared that clinicians’ sense of responsibility would be reduced and that complex burns might not be identified. The latter was regarded as the risk with the most severe consequences across all clinicians, alongside that of severe burns not being identified. By contrast, first contact clinicians were less concerned with the risk of minor burns being diagnosed as minor or insignificant.

That first contact clinicians show a widespread acceptance of the intention to use automated burn diagnosis is well in line with previous research on the use of clinical AI in general, where 77% of the respondents from different parts of the world indicated being willing to use clinical AI if needed [33]. Perceived usefulness has already been suggested as a strong predictor of both behavioral intention and technology acceptance. This is true for technologies such as smart health care [34,35], whereas in the context of burns, it has been shown for the acceptance of mHealth [21].

As the model used does not pay attention to risk perception per se in the determination of intention to use, our results can shed light on it being a potentially underlying source of influence on the intention to use. Indeed, first contact clinicians’ appraisal of the risk is more multifaceted than that of their specialized peers. A first explanation could be self-confidence, doubting their ability to interpret the output of an automated burn diagnosis [33]. Another one could be minimal experience of mHealth for burn diagnosis, while experience of the like is known to be associated with a decrease in perceived risks in the use of smart health care, thanks to technology transfer knowledge [35]. A third one could be a sense of loss in personal usefulness, first contact clinicians being more prone to answer positively to the fact that AI would replace them.

### Strengths and Limitations

This is the first study performed in resource-poor settings specifically on the topic of acceptance of automated diagnostic acceptance among health care professionals. This can complement the work done on a similar population on the acceptance of mHealth for burn injuries [21], but also results in the acceptance of AI-based diagnosis among medical students, also performed in resource-poor settings [36]. Studies on intention to use are important to be performed for specific contexts and populations of interests as it has been shown previously that physicians from resource-poor settings may have different opinions regarding clinical AI than their counterparts from other regions of the world [33]. The perceptions might also depend on the qualification of the surveyed professional [21,33,35]. The development of new methodologies to analyze the obtained data (such as machine learning techniques) might also assist in the future in targeting more specifically what might influence the intention to use new technologies by survey participants. The study also focused on 7 different risks that are relevant for the diagnosis of burn care and for which the knowledge around clinicians’ perceptions is important both for the development of algorithms as well as for implementation. It is likely that burns specialists, sharing a familiarity with the condition and aware of the progress made in assisted diagnosis are more inclined to trust the technology [37] and that, by contrasts to first contact clinicians, are more heterogeneous as a group as regards their knowledge and experience about burns, their skills and competences [21]. The sample was conveniently selected among clinicians attending 2 specialized conferences in South Africa for their interest in burn care and likelihood of using automated diagnosis would it be developed. This, however, comes with the limitation that the pool of people who can be included is relatively small, and the results cannot be generalized to other specialties or settings where the viewpoints of the professionals might differ. Furthermore, there might have been respondent bias with those accepting to fulfill the questionnaire having stronger opinions in favor of or against automated diagnosis for burns than those who did not respond. This study was cross-sectional and only assessed the intention to use a potential tool rather than effectively measuring the clinicians’ behaviors once automated diagnosis was implemented. While this information will be interesting to investigate in the future, the knowledge gathered in this study can further assist in the development of the algorithms themselves to respond most appropriately to the stakeholders’ demands. It could also be of interest to assess users’ perceptions with regard to different methodologies with varying performances, in particular when current advances in the technological domain are extremely promising.

### Future Research

This study has implications for future research as the AAM model contributes poorly to informing what the determinants of the intention to use are beyond the perceived usefulness. There is a need to assess the input of clinical risk perceptions: this should be an integral part of the model in the future, and it should receive attention during the evaluation and implementation process. Furthermore, with the body of evidence growing on the potential for automated diagnosis in burn care and its potential benefit in resource-poor settings, research on the attitudes of medical professionals with regard to this technology is necessary for a successful implementation. Finally, it would be interesting to investigate, when implementing such a technology in practice, whether it would enhance the clinical abilities of first-contact clinicians, as well as improve diagnostic efficiency, given the current state of resources and the lack of specialist clinicians.

### Conclusions

The results show that most first contact clinicians were inclined to seek automated advice for burn diagnosis if it was available. The conceptual model proposed contributes moderately to explaining what influences intention to use beyond perceived usefulness, with implications for clinical practice where the perceived usefulness makes little doubt. This is encouraging as there are good reasons to believe that the performance of automated diagnostic support will continuously improve. When seeking additional determinants, greater attention should be placed on clinical risk perceptions as well as negative attitudes due to bad experiences or unrealistic expectations. These dimensions should also naturally belong to any AI implementation process in practice, not least to ensure sustainability.

## Supplementary material

10.2196/56300 Multimedia Appendix 1 Table S1. CHERRIES checklist. CHERRIES: Checklist for Reporting Results of Internet E-Surveys.

## Abbreviations

AAM: Automation Acceptance Model
AI: artificial intelligence
EMSSA: Emergency Medicine Society of South Africa
