# Supplementary material for: Intention to Use Automated Diagnosis and Clinical Risk Perceptions Among First Contact Clinicians in Resource-Poor Settings: Questionnaire-Based Study Focusing on Acute Burns
Source: JMIR Hum Factors. 2025 Jun 3;12:e56300. doi: 10.2196/56300 (PMC12151446; doi:10.2196/56300)
Supplement: Multimedia Appendix 1 [file humanfactors-v12-e56300-s001.pdf]

Table S1. CHERRIES Checkist[1].

Item Category	Checklist Item	Explanation
Design	Describe survey design	The study was cross-sectional, with the population described as a convenient sample of participants to two medical conferences.
IRB approval and informed consent process	IRB approval	The study was approved in South Africa by the Human Research Ethics Committee of the University of Cape Town (Dnr 823/2019) and in Sweden by the Ethics committee (Etikprövningsmyndigheten (Dnr 2019-05122)).
	Informed consent	All participants gave their informed consent on the first page of the survey.
	Data protection	No personal information was collected for the purpose of the study, and all the data collected is stored in safe location only accessible to the research team.
Development and pre-testing	Development and testing	The questionnaire was developped in three parts and based on previously existing questionnaires related to the Automation Acceptance Model, as well as perceived risks related to the use of automated diagnosis for burn injuries. It was piloted tested for both content and timing estimate prior to distribution.
Recruitment process and description of the sample having access to the questionnaire	Open survey versus closed survey	The survey had open settings but it was distributed in closed environments.
	Contact mode	Participants to the conferences were made aware of the survey during their registration to the conference, but the survey link was provided at the conferences.
	Advertising the survey	In addition to information about the survey being provided to the participants of the conferences at registration, pamphlets were distributed around the conference sessions.
Survey administration	Web/E-mail	The survey was performed on the RedCap website and accessed through a QR code provided
	Context	The website contained exclusively the survey.
	Mandatory/voluntary	The survey was voluntary
	Incentives	There were no incentives to participate
	Time/Date	The data were collected during two conferences happening in South Africa in 2019: The meetings were *the Pan African and South African Burn Congress 2019* and *the 7th Emergency Medicine Society of South Africa (EMSSA) International Conference*.

	Randomization of items	Yes
	Adaptive questioning	No
	Number of Items	Between 9 and 16 items were presented per page
	Number of screens (pages)	There were 8 screens with quetsions
	Completeness check	Participants had to finish a section to get to the next one.
	Review step	Yes participants could access their questions again before submitting
Response rates	Unique site visitor	We did not assess the number of site visitors, or of conference participants who got access to the survey.
Preventing multiple entries from the same individual	Cookies or IP checked	No methodologies were used to prevent multiple entries. We can however notice from the demographic entries that no individual participated twice.
Analysis	Handling of incomplete questionnaires	Only sections of questionnaires that were completed were used. Others were disregarded.
	Questionnaires submitted with an atypical timestamp	All questionnaires filled in were included, irrespective of the time taken to be filled. Overall the questionnaire took approximately 5 minutes.
	Statistical correction	No weighting of items or propensity score were perfomed.
[1] Checklist described in Eysenbach G. Improving the Quality of Web Surveys: The Checklist for Reporting Results of Internet E-Surveys (CHERRIES). J Med Internet Res 2004;6(3):e34		
